# Mobile Sensing and Support for People With Depression: A Pilot Trial in the Wild

**DOI:** 10.2196/mhealth.5960

**Published:** 2016-09-21

**Authors:** Fabian Wahle, Tobias Kowatsch, Elgar Fleisch, Michael Rufer, Steffi Weidt

**Affiliations:** ^1^ ETH Zürich Department of Management, Technology and Economics Zürich Switzerland; ^2^ University of St Gallen Institute of Technology Management St Gallen Switzerland; ^3^ Department of Psychiatry and Psychotherapy University Hospital Zürich University of Zürich Zürich Switzerland

**Keywords:** depression, mHealth, activities of daily living, classification, context awareness, cognitive behavioral therapy

## Abstract

**Background:**

Depression is a burdensome, recurring mental health disorder with high prevalence. Even in developed countries, patients have to wait for several months to receive treatment. In many parts of the world there is only one mental health professional for over 200 people. Smartphones are ubiquitous and have a large complement of sensors that can potentially be useful in monitoring behavioral patterns that might be indicative of depressive symptoms and providing context-sensitive intervention support.

**Objective:**

The objective of this study is 2-fold, first to explore the detection of daily-life behavior based on sensor information to identify subjects with a clinically meaningful depression level, second to explore the potential of context sensitive intervention delivery to provide in-situ support for people with depressive symptoms.

**Methods:**

A total of 126 adults (age 20-57) were recruited to use the smartphone app Mobile Sensing and Support (MOSS), collecting context-sensitive sensor information and providing just-in-time interventions derived from cognitive behavior therapy. Real-time learning-systems were deployed to adapt to each subject’s preferences to optimize recommendations with respect to time, location, and personal preference. Biweekly, participants were asked to complete a self-reported depression survey (PHQ-9) to track symptom progression. Wilcoxon tests were conducted to compare scores before and after intervention. Correlation analysis was used to test the relationship between adherence and change in PHQ-9. One hundred twenty features were constructed based on smartphone usage and sensors including accelerometer, Wifi, and global positioning systems (GPS). Machine-learning models used these features to infer behavior and context for PHQ-9 level prediction and tailored intervention delivery.

**Results:**

A total of 36 subjects used MOSS for ≥2 weeks. For subjects with clinical depression (PHQ-9≥11) at baseline and adherence ≥8 weeks (n=12), a significant drop in PHQ-9 was observed (*P*=.01). This group showed a negative trend between adherence and change in PHQ-9 scores (rho=−.498, *P*=.099). Binary classification performance for biweekly PHQ-9 samples (n=143), with a cutoff of PHQ-9≥11, based on Random Forest and Support Vector Machine leave-one-out cross validation resulted in 60.1% and 59.1% accuracy, respectively.

**Conclusions:**

Proxies for social and physical behavior derived from smartphone sensor data was successfully deployed to deliver context-sensitive and personalized interventions to people with depressive symptoms. Subjects who used the app for an extended period of time showed significant reduction in self-reported symptom severity. Nonlinear classification models trained on features extracted from smartphone sensor data including Wifi, accelerometer, GPS, and phone use, demonstrated a proof of concept for the detection of depression superior to random classification. While findings of effectiveness must be reproduced in a RCT to proof causation, they pave the way for a new generation of digital health interventions leveraging smartphone sensors to provide context sensitive information for in-situ support and unobtrusive monitoring of critical mental health states.

## Introduction

In October 2012, the World Health Organization (WHO) estimated that 350 million people worldwide suffer from depression [1]. It is expected that depression will be the world’s largest medical burden on health by 2020 [2]. Beyond its burden on society, depression is associated with worse global outcomes for the affected individual, including reduced social functioning, lower quality of life in regards to health, inability to return to work, as well as suicide [3]. Traditionally, depression is treated with medication and/or face to face psychotherapy using methods such as cognitive-behavioral therapy (CBT), which has been proven to be effective [4]. However, it must be noted that mental health personnel, usually psychologists and psychiatrists with a specialized education that goes beyond both geospatial ubiquity and skills of general practitioners, are strongly required for CBT but limited. For 50% of the world’s population there is only one mental health expert responsible for 200 or more people [2]. In recent years, this led to the rise of digital versions of CBT in the form of educational interactive websites and smartphone apps [5]. Many of these solutions presented reasonable effects sizes [6], sometimes even on a par with face to face therapy [7]. However, a recent review revealed an array of shortcomings still present in most of the approaches, for example, the lack of personalization and missing in-situ support [8]. A key to the solution could lie in digital health interventions offered through modern smartphones and their sensors. The overwhelming prevalence of smartphone devices in society suggests that they are becoming an integral part of our lives. Recent estimates indicate that, for example, 64% of American adults and almost one quarter (24.4%) of the global population own a smartphone [9]. By 2016, the number of global smartphone users is estimated to reach 2.16 billion [10]. With these devices, an ensemble of techniques from the field of Artificial Intelligence, mobile computing, and human–computer interaction potentially represents the new frontier in digital health interventions. Learning systems could adapt to subject’s individual needs by interpreting feedback and treatment success [11] and smartphones could provide important context information for adequate in-situ support [12,13], in the form of interactive interventions and even infer a subject’s condition state. For example, physical activity, shown by numerous studies to be related to depression [14,15], can be approximated by acceleration sensors [16], duration, and time of the day of stays at different physical locations were shown to be related to a person’s mental state and can be approximated by WiFi and global positioning systems (GPS) information [17,18]. Another relevant aspect is social activity. It is highly related to a subject’s mental state and the risk of developing depression [19,20]. Smartphones offer numerous sources of information acting as proxies for social activities such as the frequency and average duration of calls, or the number of different persons being contacted.

While until today, there is no study presenting results of a context aware digital therapy for people with depression providing in-situ support, recent studies by Saeb et al and Canzian et al [17,18] demonstrated promising results in objectively and passively detecting whether a subject might suffer from depression solely using information provided by the smartphone. Saeb et al [17] used the information of GPS sensors and phone use statistics to distinguish people without (Patient Health Questionnaire, PHQ-9, <5) from people with signs of depression (PHQ-9≥5) in a lab experiment over 2 weeks with high accuracy. Canzian et al [18] were able to show a tendency of correlation between a range of GPS metrics similar to the ones presented by Saeb et al [17] and a self-reported depression scores. In another recent explorative study, Asselbergs et al [21] were able to resemble a subject’s day-to-day mood level solely based on passively collected data provided by the smartphones with 55% to 76% accuracy. This development shows a promising direction in objective and unobtrusive mental health screening, potentially reducing the risk of undetected and untreated disorders. It is, however, still an open question whether it is possible to distinguish people with and without clinically relevant depressive symptoms (PHQ-9≥11 [22]) in an uncontrolled real life scenario.

This would open up a range of opportunities for unobtrusive mental health screening potentially able to alert a subject if a critical mental state is reached and, as a consequence, an additional professional treatment highly recommended. This could not only reduce costs in the health care system by preventing severe cases from getting into worse and costlier states, but also by preventing subjects with symptom severity below clinical relevance to strain the system.

Therefore, the aim of the present work was to explore the potential of context-sensitive intervention delivery to provide in-situ support for people with depressive symptoms, and to explore the detection of daily life behavior based on smartphone sensor information to identify subjects with a clinically meaningful depression level.

## Methods

### System Architecture

At the core of the present work, a novel digital health intervention for people with depressive symptoms was developed.

**Figure 1:**
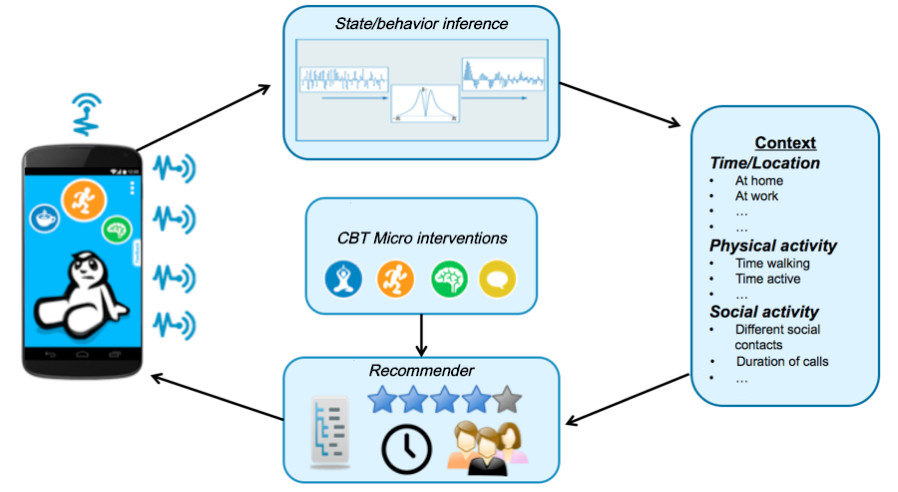
Schematic overview of Mobile Sensing and-Support (MOSS) app process flow. Note: Starting left (1) MOSS app collects sensor and use data, (2) data is analyzed and transformed into (3) context information, (4) context information in combination with user preference and decision logics are used to recommend (5) evidence-based interventions presented via (1) the MOSS app.

Figure 1 represents a schematic overview of the process flow within the Mobile Sensing and Support (MOSS) app. A range of smartphone sensors holds potentially valuable information about a subject’s individual context. Using techniques from the field of machine learning, this sensor information can be used to infer a subject’s behavior. For example, classification techniques [23] can be used on accelerometer and GPS data to detect what type of physical activity a subject carried out throughout the day or how much time the subject spent at home or outside. These analyses result in an array of context features the app uses to provide the subject with evidence-based interventions stemming from the theory of cognitive behavioral therapy. After each intervention, the system receives passive or active feedback from the subject regarding the last recommendation. Over time, this enables the system to learn a subject’s preference to change recommendations accordingly.

In the following sections, we give a detailed description of the context features, the functional principles of the recommender algorithm, as well as a description of the developed interventions.

### Context Features

In order to be able to provide a subject with meaningful recommendations in everyday life, we need to analyze subjects’ context solely based on their interaction with a smartphone. In a first step, the current implementation constructs a context from information about time of the day, location, smartphone usage, and physical and social behavior. While information such as time of the day or smartphone usage can directly be extracted, other information needs to be approximated with the help of behavioral proxies derived from processed sensor data. For sensor data collection, we made extensive use of the open source framework UBhave by Hargood et al [24]. Next, we provide an overview of context features that we developed for the study together with a motivation why the feature is relevant in the context of depression, followed by a detailed description how our recommendation algorithm uses these features to present meaningful interventions.

#### General Activity

Numerous studies showed a bidirectional relationship between depressive symptoms and physical activity [25-27]. Our approximation of physical activity is 2-fold. Using the acceleration sensor data provided by the smartphone, we analyze a subject’s general activity level and a subject’s walking time.

To assess the general activity levels, the standard deviation of the three-dimensional (3D) acceleration norm was computed according to Equation 1:

STDEV(3DaccNorm) = STDEV(√(a_x_^2^+a_y_^2^+ a_z_^2^ )– 9.81m/s^2^) (1)

Where a_x_^2^, a_y_^2^ and a_z_^2^ represent the 3 acceleration axis and 9.81m/s^2^ represents the gravity of Earth.

Each acceleration axis was sampled with 100 hz resulting in a total of 300 samples per second. To estimate a subject’s general activity intensity over a finite time window, the standard deviation of the 3D acceleration norm was computed as described by Vähä‐Ypyä et al [28]. A recent study showed that the standard deviation of the 3D acceleration norm resembles intensity of physical activity of 2 widely used commercial acceleration-based activity trackers with reasonable consensus [29]. For this trial, we used a time window of 2 minutes. As we did not aim at classifying micro movement, this window size was appropriate for our app needs and trades of phone memory usage and frequency of computation and information gain.

#### Walking Time

To approximate the walking time, for every time window of 2 minutes, we made use of the standard deviation of the 3D acceleration norm (1) again. Adapting the approach of Vähä‐Ypyä et al [28], we used an intensity-based classification approach to determine whether a subject was walking. To derive a meaningful threshold for our app, we conducted numerous tests with different test subjects varying walking speed and smartphone carrying positions. We found, that this approach is robust to variance in the orientation of the smartphone was confirmed by Kunze and Lukowicz [30] and different walking speeds. We chose the final threshold at 1.5.

#### Time at Home

To measure the time a subject stays at home, an approach by Rekimoto et al [31] for WiFi-based location logging was adapted. Every 15 minutes, the WiFi basic service set identifier of hotspots in the surrounding were scanned. Based on a rule-based approach, MOSS tried to learn a subject’s home by comparing WiFi fingerprints stored during the first 3 consecutive nights. If a reasonable overlap of hotspots was detected, the MOSS app stored this information. In order to avoid tagging the wrong location, the MOSS app asked the subjects whether they are at home, if the tagged fingerprints were not detected in any 3 consecutive nights.

#### Phone Usage

This feature measured the total time subjects were using their mobile phone depicted by the time the smartphone was unlocked following Saeb et al [17]. The time spent with the MOSS app was excluded.

#### Geographic Movement

As described earlier, 2 recent studies were able to show a relationship between depressive symptoms and geographic movement. Building on these works, an array of metrics from GPS information were constructed [17,18]. Every 15 minutes, coordinates of the current location of the subject were captured. From these coordinates, the maximum and the total distance traveled were calculated using techniques for geographical distance calculation. Additionally, the location variance was calculated from the latitudes and longitudes using the Equation 2:

locVar = log(σ_lat_^2^ + σ_long_^2^) (2)

To compensate for skewness in the distribution of location variance across participants, we also used the natural logarithm of the sum of variances.

#### Number of Unique WiFi Fingerprints

In addition to GPS information as a proxy for geographic movement, every 15 minutes, WiFi fingerprints of the surrounding were scanned. Besides the fingerprints for home detection, a list of unique hotspots was kept to keep track of the total number of fingerprints detected.

#### Number of Text Messages

This feature kept track of the incoming and outgoing text messages together with a count of different unique contacts the messages were sent to and received from. This adopts a social mining approach by Eagle et al [32] and represents one dimension of social activity. Past studies showed a negative correlation between the amount of social interaction and depression levels [19] and diminished social activity in increased depression levels [20].

#### Number of Calls

This feature kept track of the number of incoming and outgoing calls a subject made together with a count of different individuals as also described in [32]. This feature follows the same argumentation line as the number of text messages feature.

#### Number of Calendar Events

This feature kept track of the number of calendar events stored. It distinguished between events taking place in the morning, afternoon, and evening time [33]. This feature tried to act as a proxy for stress caused by too many calendar events, which could have an influence on depression levels [34]. Further, calendar events in the evening could represent another dimension for social activity (eg, a cinema or restaurant visit). As we solely look at the number of events per time frame, we cannot interpret the context of the event.

**Figure 2:**
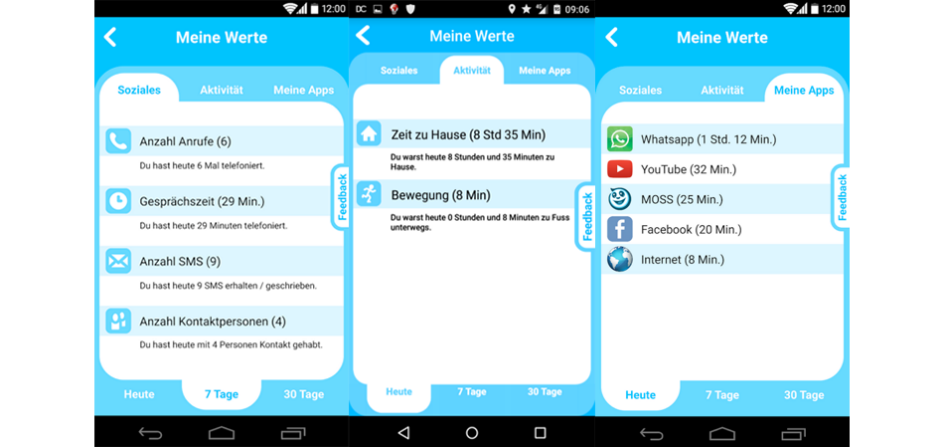
Social activity, physical activity, and used app screens of the Mobile Sensing and Support (MOSS) app. Note: The initial user interface was in German, the first screen shows number of calls, total time of calls, amount of SMS text messages, and number of persons contacted over the last 7 days. The second screen shows time spent at home and time spent moving during the current day.

To provide subjects with insights about their behavior and to further guarantee a high level of transparency about the collected data and computed features, we implemented a dedicated section into the user interface. Here, the subject was able to observe collected information over the course of different time periods. The screens of Figure 2 provide the subcategories social activity, physical activity, and used apps.

### Recommender

The recommender was responsible for presenting interventions to the subject. It tried to optimize the delivered content with respect to the context and subject preferences.

As described earlier, the context was composed of time of the day, the location, smartphone usage, as well as physical and social behavior. The recommender was designed to work in 2 phases. In the first phase it delivered interventions based on assumptions about the behavior of the general depressed population and handcrafted weights for appropriate interventions to be delivered depending on the characteristics of the context (this will later be explained in more detail). In the second phase, the delivery quality was enhanced by adjusting the assumptions according to a subject’s actual behavior. In Table 1, example assumptions about the general depressed population and the characteristics of a subset of context features are presented.

**Table 1:** Assumptions about people with depression.

Context feature	Weakly pronounced per day	Strongly pronounced per day
Time spent at home	Weekdays <7 hours a day	Weekdays >14 hours a day
Total number of calls	0 calls per day	>6 calls per day
Walking time	Weekdays <30 minutes	Walking time >300 minutes

Only context features where reasonable assumptions of characteristics in the overall population could be made, were included into the recommendation algorithm. Furthermore, this includes the number of texts sent/received, number of calendar events, average call duration, and time of phone use.

To reduce complexity, interventions with similar characteristics were grouped into baskets. For each basket, domain experts, in our case 2 trained psychologists, attached importance weights of features, in order to help the MOSS app to decide which baskets should be considered for recommendation depending on the subject’s context. For example, the recommendation to take a walk in the park should be related to the general activity level of a subject so that, if the subject had a low general activity, the probability that a walk in the park is recommended, increases. The score for each basket is calculated according to Equation 3:

basketScore_n = w_1_ * scaleToRange(x_1_max_, x_1_min_, x_1_) + w_2_ * scaleToRange(x_2_max_, x_2_min_, x_2_)+ … + w_n_ * scaleToRange(x_n_max_, x_n_min_, x_n_) (3)

Where w_n_ is the weight of feature n, x_n_ is the value of feature n over the last 24 hours, scaleToRange() is a function to calculate the fraction of x_n_ reached of the range between defined small and large values of x_n._

**Figure 3:**
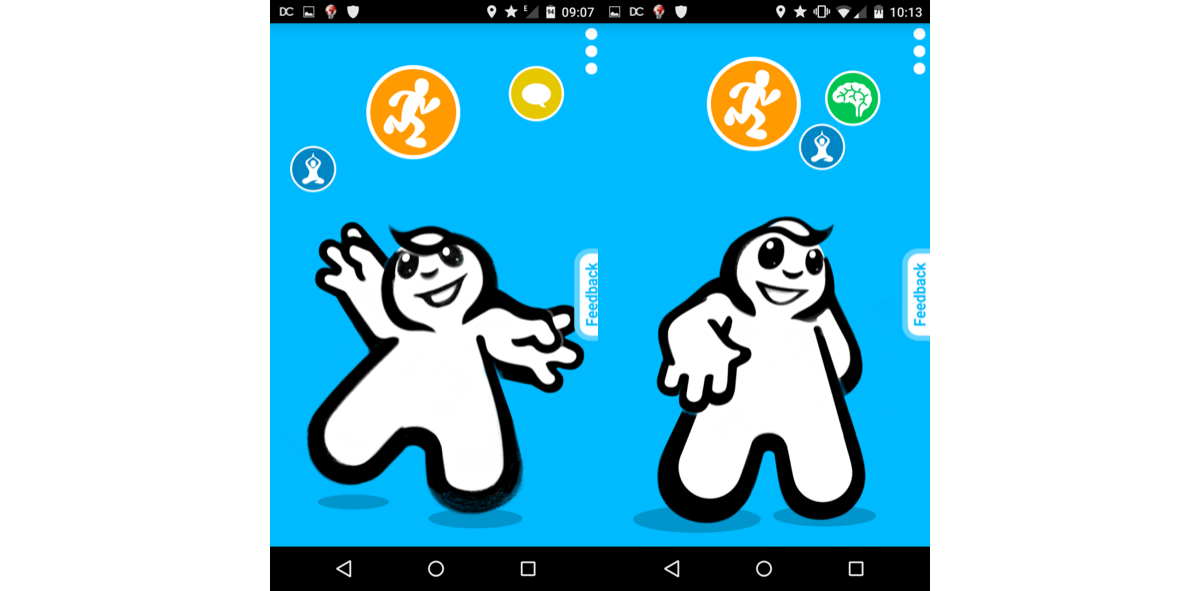
Example recommender results with physical activity baskets showing the highest score (orange circle) compared with social activity (yellow), mindfulness (green), and relaxation (blue).

The baskets with the highest scores were presented to the subject in the form of touchable circles on MOSS’s home screen as shown in Figure 3. The size of the circles indicated the recommendation score of the basket. The higher the score, the larger the radius of the circle. Unique icons represented the type of the domain the basket belongs to. Figure 3 shows examples where baskets with physical exercises received the highest score (orange circle).

**Figure 4:**
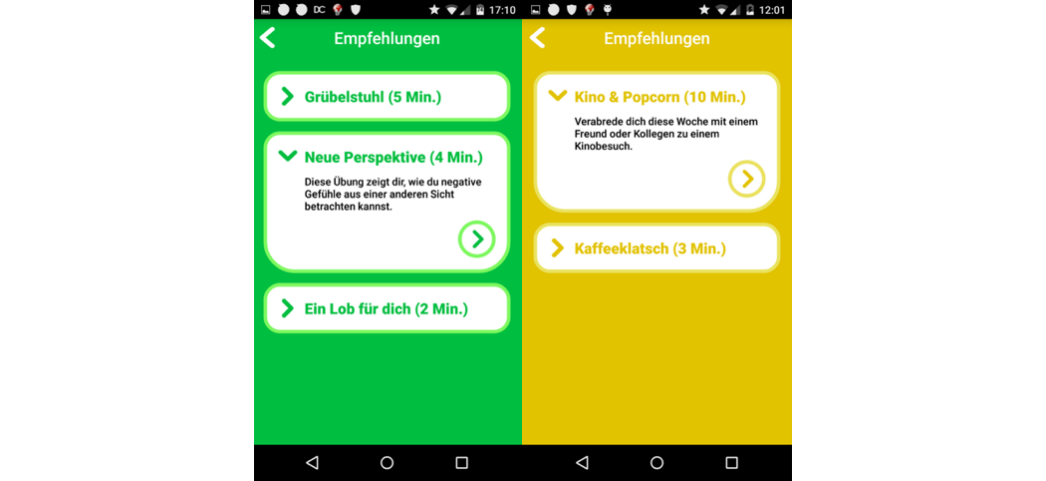
Sample screenshots of lists of interventions of two different baskets. Each item shows the approximate time it takes to carry out the intervention together with a short summary (in German language) Note: The left, green list presents 3 mindfulness exercises: “muse chair,” “new perspective,” and “praise yourself.” The right, yellow list presents 2 social exercises: “Movies&Popcorn” and “kaffeeklatsch.”.

This basket score computation was repeated every 6 hours to present relevant baskets. Once the subject clicks an icon, specific interventions of the related basket are presented to the user. See Figure 4 for an example of interventions of a chosen basket presented to the user.

For the most relevant baskets of each domain and every 6 hours, only the top 3 interventions can be carried out by the user. Once the user completes/neglects all 3 interventions, the basket and its related circle disappears from the home screen until the next context evaluation. In order to determine which 3 interventions of each basket are shown to the user, individual interventions are ranked according to a score.

**Figure 5:**
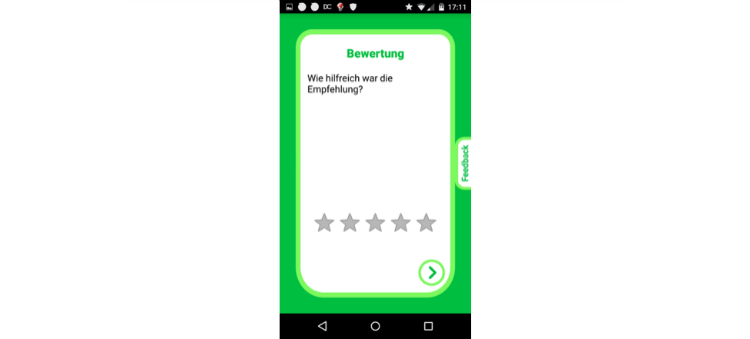
Sample screenshots of an intervention rating. The subject is asked for perceived usefulness of the intervention on a 5-star rating scale (in German language).

The following Equation (4) was used to score interventions using a weighted combination of the subject’s preference depicted by a simple star rating after the execution of an intervention (Figure 5), the completion rate of the interventions depicted by the fraction of times the subject finished an intervention and did not cancel it early and a small factor of chance:

interventionScore = 0.75 * pastRatings/5 – 0.25 * cancelationRate + 0.5 (if random ≤ 0.05) (4)

Where pastRatings is the average rating over all past ratings for this intervention, cancelationRate is the fraction of times the subject canceled the intervention early and random is a uniformly distributed random number between zero and one.

The static weight parameters of the intervention score were set following an explorative approach. The values follow the assumption that past ratings of an intervention represent the preference for an intervention and therefore should have the highest impact on the scoring function. Contrary, the cancelation rate has a negative impact on the overall score. The decision to cancel an intervention early, is not necessarily related to a subject’s general liking of the intervention, therefore the impact is significantly lower than past ratings. Finally, to prevent interventions from not being recommended over a long period of time because their average past rating is too low, a factor of chance is introduced with a positive impact on the score to promote fluctuation.

In addition, 2 clinically trained psychologists predefined rules to prevent the MOSS app making unreasonable intervention recommendations. For example, an intervention asking the subject to lie down for a relaxation exercise is only recommended if the subject is at home and if the current time period is in the morning or in the evening.

Also, after each execution, an intervention was blocked for a period of time to avoid early repetition. The length of this period in hours depended on the subject’s rating of the intervention according to Equation 5:

blockTime = 36 * (6 – pastRating) (5)

Where pastRating is the last rating of the intervention.

In the second phase, the following changes to Equation 3 were applied. After 2 weeks, the basket scoring computation (see basket score Equation 3) was automatically adjusted, by applying information of individual subject’s actual behavior: x_n_max_ and x_n_min_ are defined as μ ± (2*σ) (ie, the average feature value of the subject during the last week ±2 times the standard deviation). This way, the MOSS app does not suffer from potentially flawed assumptions about a subject’s behavior with respect to the general population and adapts to the subject’s actual behavior.

### Interventions

In line with the majority of Web-based health interventions targeting people with depressive symptoms [8], MOSS uses CBT. CBT is a highly-structured psychological treatment [35]. It is based on the assumption that thoughts determine how one feels, behaves, and physically reacts. This form of intervention contains various treatments using cognitive and behavioral techniques with the assumption that changing maladaptive thinking leads to change in affect and behavior. Examples for therapeutic CBT interventions are activity scheduling, relaxation exercises, cognitive restructuring, self-instructional training, or skills training such as stress and anger management [36]. CBT is often regarded as the mental health intervention of choice due to its large evidence on a variety of psychological disorders [4]. Moreover, and with regard to its structure, it is suitable for implementation in digital health interventions [37-40]. For MOSS, a set of 80 interventions including social, relaxation, thoughtfulness, and physical activity exercises were designed and implemented following best practice in CBT.

To promote motivation and adherence [41], 8 different types of diverse interactive interventions were used. Table 2 provides an overview of different types of interactive interventions together with a specific example. Figure 6 depicts exemplary screenshots of the MOSS app.

**Table 2:** Overview of interactive elements of the Mobile Sensing and Support (MOSS) app.

#	Type of intervention	Description	Example
1	Activity tracker	Based on the walking detection described above, every 2 minutes the progress is updated.	“Take a 10 minute walk outside”
2	Quiz	The subject is asked to answer questions about educational material shown before. Answers can be chosen from multiple choice answers.	“How do you define awareness?”
3	Checkbox	The subject is asked to tick a box with a checkmark on the screen.	“Think of something you did well during the last days, if you found something, check the box!”
4	Button	The subject is asked to tap a virtual button on the screen decreasing a countdown to (eg, encourage physical exercise).	“Morning exercise: sit on the edge of your bed, place the phone on your lap and tap the countdown button with your nose 5 times”
5	Mirror	The subjects see themselves on the smartphone, using the frontal camera. After a countdown the camera is switched off.	“You will see yourself on the phone. Look at yourself in the eye and smile for at least 20 seconds”
6	Audio	Audio files are played to the subject. The subject can pause/stop the audio with common controls.	“Press the play button and listen to instructions for a breathing exercise”
7	Multitext	Educational texts are presented to the subject, spanning multiple screens the subject can navigate through.	“On the following 3 pages, you will get an introduction on awareness”
8	Countdown	The subject is asked to carry out a distinct task during a given time. After the countdown ends, a signal sound rings.	“Sit straight on a chair, start the countdown, lift your feet from the ground and hold this position until you hear a signal sound”

**Figure 6:**
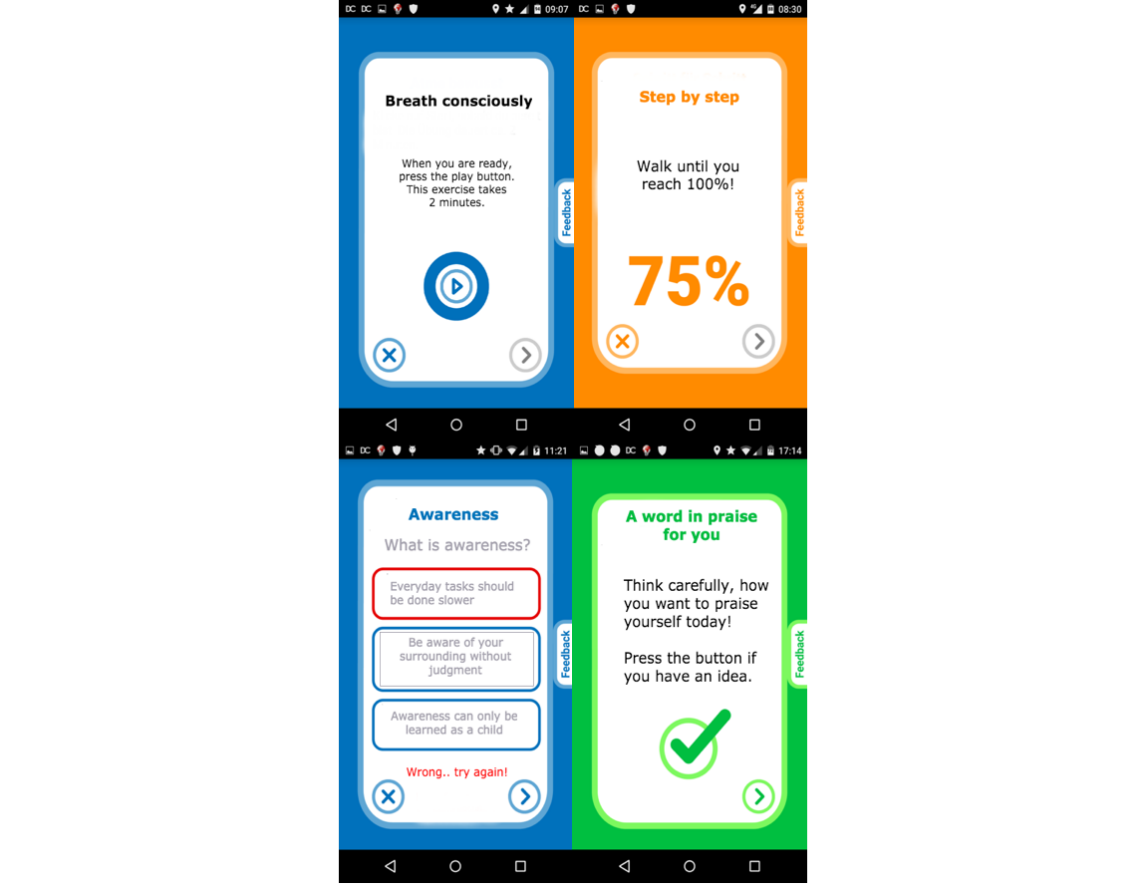
Sample screenshots of intervention types (see Table 2) #6, #1, #2, and #3 (clockwise). Note: The screenshots were translated from German for demo purpose.

### Trial Design

A monocentric, single-arm clinical pilot study was conducted. The study was approved by the local ethics committee of the Canton of Zurich in Switzerland and the Swiss Agency for Therapeutic Products. It was conducted in full accordance with the Declaration of Helsinki, with all subjects providing their electronic informed consent prior to participation. As the main interest lied in a proof of concept of the proposed MOSS app, emphasis was put on real life conditions. A range of different recruitment channels was used to attract subjects from the general public; they included physical flyers, Internet posts on relevant Web-based bulletin boards, and the Google Play Store. Interested people were lead to a website with information about the project and an initial screening survey. To be applicable for the study, subjects had to be at least 18-years old, not suffering from bipolar disorder, addiction, or suicidality. If subjects met no exclusion criteria they received a participation code and a download link to the MOSS app. At no point, direct contact with members of the research team was necessary. Subjects were able to enroll on a rolling basis until 2 weeks prior to the end of the trial. The clinical trial took place within 9 months, from January 2015 until September 2015.

### Analysis

#### Symptom Severity Change

As we were interested in changes of PHQ-9 scores of subjects while using the MOSS app, we compared PHQ-9 scores after different time-period lengths. A Kolmogorov-Smirnov test [42] rejected the normality assumption, we therefore conducted Wilcoxon signed rank tests at time t_0_ and t_n_. In order to be able to do group-wise tests, we synchronized the starting time point t_0_ among all subjects and repeated tests between t_0_ and t_n_. N is incremented for every 2 weeks where subjects were still participating and provided a PHQ-9 value. We included subjects who were considered clinically depressed (PHQ-9≥11) at baseline measurement and who at least used the MOSS app for consecutive 4 weeks and provided 2 PHQ-9 measures after the baseline. We considered 2 additional measurements the minimum in order to conduct reasonable analysis.

#### Relationship of MOSS App Usage and Severity Change

Even though causation cannot be tested with the study design, we tried to find evidence that cumulated change in symptom severity is related to MOSS usage. As a proxy, we used the number of times MOSS was used. A single app use was defined as at least one intervention execution within a session. Multiple intervention usage within one session does not count as multiple MOSS usage. To quantify the relationship between cumulated change in symptom severity and MOSS usage, we conduct a Spearman correlation analysis between the total number of MOSS Sessions and the absolute change in PHQ-9 level between t_0_ and t_end_. Spearman correlation was used because both distributions deviated from normality (*P*<.001, Kolmogorov-Smirnov test).

#### Passive Depression Detection

This section describes the development of MOSS’s depression detection model from features that are derived from smartphone sensor information.

As described earlier, we developed an array of features acting as proxies for behavioral dimensions potentially related to depression. We proposed that a combination of these feature characteristics act as the base for a depression detection model.

For each of the features outlined above, we calculated descriptive statistics over the course of 14 days prior to each time a subject provided a new PHQ-9 measurement. This adds additional potentially valuable information with respect to our classification goal and includes the following computations: mean, sum, variance, minimum, and maximum values per day of the last 2 weeks. In total, this leads to a feature space of 120 features potentially holding information about a subject’s depression level of the last 2 weeks. The goal therefore is to relate these time-dependent feature characteristics, to a subject’s current depression level. In a very first step, the developed model aimed at separating subjects into 2 groups. For this, we chose a PHQ-9 cut off value of 11, in line with the PHQ-9 [22] to separate people with (≥11) from people without (≤10) a clinically relevant depression level. In order to derive a binary classification model, we make use of techniques from supervised machine learning. In particular, 2 learning algorithms were used; Support Vector Machines (SVM [43]) and Random Forest Classifier (RFC [44]), which share a predominant role in a range of research domains [45].

The SVM is a supervised learning model with associated learning algorithms that analyze data used for classification analysis. The concept of the SVM method is to project the input features onto a high dimensional space using the kernel-method. In this space, based on transformed feature values, a set of hyper planes is constructed. The goal of the SVM method is to generate optimal hyper planes that are used as decision boundaries to separate different classes. In our system, the radial basis function (RBF) kernel was used for mapping the features to a multidimensional space. SVM and kernel parameters were optimized using Nelder-Mead simplex optimization [46,47].

RFC is a classification algorithm that uses an ensemble of decision trees [48]. To build the decision trees, a bootstrap subset of the data is used. At each split the candidate set of predictors is a random subset of all predictors. Each tree is grown completely, to reduce bias; bagging and random variable selection result in low correlation of the individual trees. This leads to the desirable properties of low bias and low variance [49].

To report on classification performance of the model proposed for this study, we make use of accuracy scores. Accuracy is defined as the fraction of correctly classified samples of both, positive and negative classes. This makes it easy to interpret and ensures a neutral interpretation with respect to importance of positive and negative classes [50].

For unbiased performance estimation of both classifiers, leave-one-out cross validation was conducted [51]. This involved splitting the data into as many subsets as subjects who provided at least one PHQ-9 value in addition to the baseline (adherence ≥2 weeks). All but one set is used to train the models. The left out set is used for testing. This procedure is repeated for every subject, providing a range of unbiased test scores. The average of these scores is reported as the unbiased performance estimate [52]. In order to provide further insights on the classification performance, sensitivity and specificity scores are also reported together with the accuracy score [50].

## Results

### Subject Statistics

A total of 126 subjects were recruited from the general public. A large portion of subjects uninstalled the MOSS app within the first 2 weeks (64/126, 50.8%). Another 20.6% subjects (26/126) uninstalled the app in the following 2 weeks. Approximately one-fifth of the subjects (28/126, 22.2%) had an adherence of 4 weeks or longer providing at least 2 PHQ-9 measures in addition to the baseline measure (male = 10, female = 18). Because the study was primarily advertised in Switzerland and on German-speaking Internet forums, the majority of participants came from Switzerland and Germany.

### Symptom Severity Change

**Figure 7:**
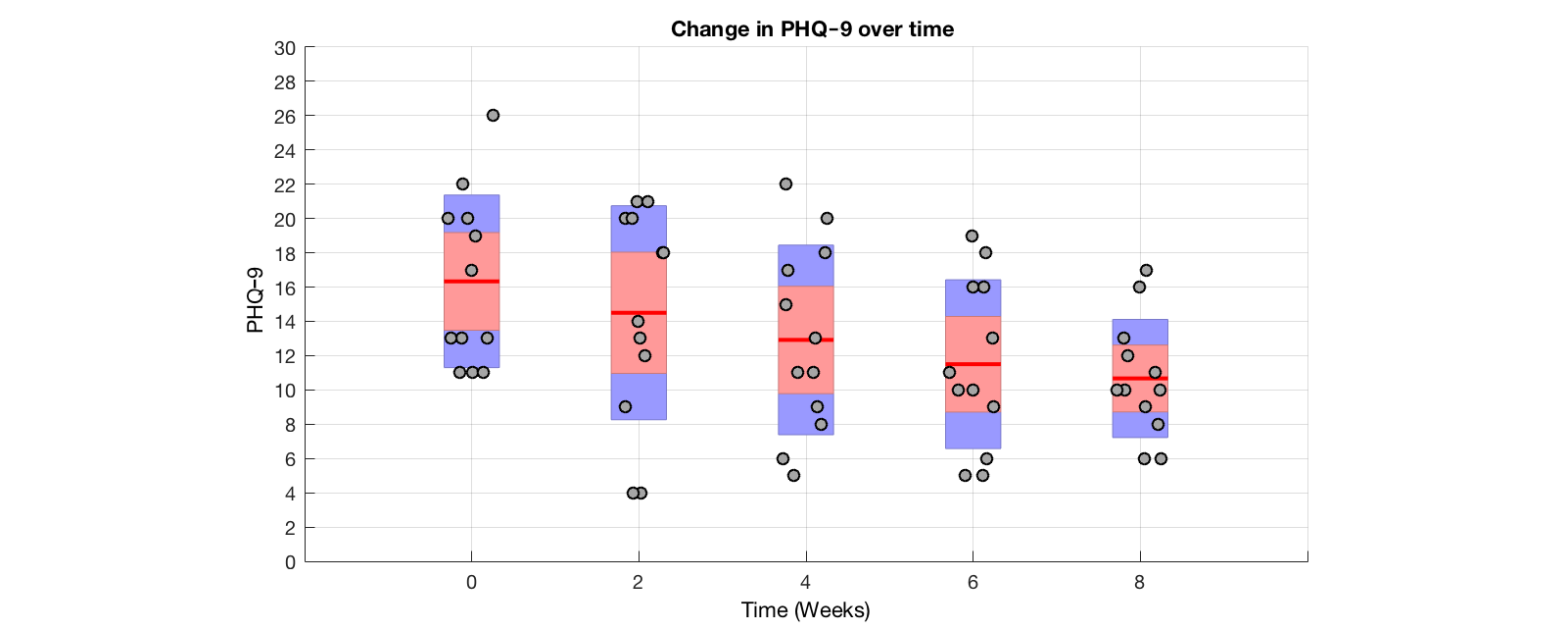
Plot of PHQ-9 progression of clinically depressed individuals over time. Note: Gray dots represent individual PHQ9 values, red lines show distribution mean for each time point, the red area shows the 95% confidence interval for the mean, the blue surface shows 1 standard deviation.

Figure 7 shows the PHQ-9 progression of subjects who were classified as clinically depressed at the first use of the MOSS app and who had an adherence of at least 4 weeks, providing 2 PHQ-9 values in addition to the baseline measure. Twelve subjects met these criteria, where all of these had an adherence of 8 weeks or longer. For every 2 weeks of MOSS app use, the PHQ-9 distribution represented by a bar plot is shown.

Table 3 provides further insights on the development of PHQ-9 values of the 12 subjects. For every 2 weeks, we present the interquartile range together with the median of PHQ-9 scores. For every additional 2 weeks, we conducted a Wilcoxon sign-rank test with respect to t_0_. At t=6 and t=8 we observe a significant difference in means.

**Table 3:** Wilcoxon signed rank test results between t and t_n_.

PHQ-9 score^a^	tn, median PHQ-9 (IQR^b^)	t0, median PHQ-9 (IQR)	N	*z* ^c^	*P* value
t2, median (IQR)	14.00 (11.25-20.00)	13.00 (11.00-20.00)	12	0.283	.77
t4, median (IQR)	13.00 (8.75-17.25)	13.00 (11.00-20.00)	12	1.216	.22
t6, median (IQR)	11.00 (8.25-16.00)	13.00 (11.00-20.00)	12	2.013	.04^d^
t8, median (IQR)	10.00 (8.75-13.75)	13.00 (11.00-20.00)	12	2.479	.01^d^

^a^^a^PHQ-9: Personal Health Questionnaire.

^b^IQR: interquartile range.

^c^Wilcoxon signed rank test.

^d^Significant at the 5% level.

Subject’s with a PHQ-9<11 at baseline and an extended time of use of at least 4 weeks (n=8) did not show significant difference between t_0_ and t_4_. (*P*=.22)

### Relationship of MOSS Usage and Symptom Reduction

**Figure 8:**
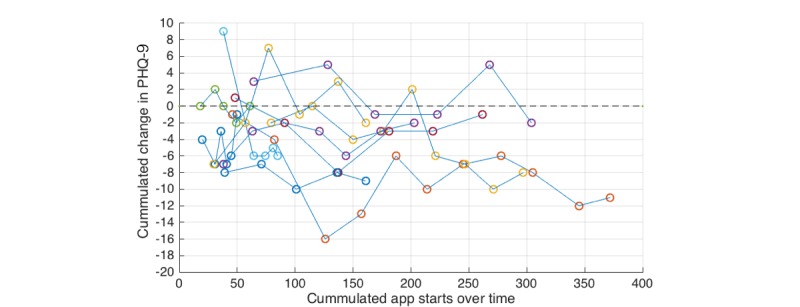
Scatter plot of cumulated app starts per subject over time and cumulated change in PHQ-9 values. Note: The development of PHQ-9 scores of individual subjects is indicated by connected points of the same color.

Figure 8 shows a scatter plot of cumulated app starts over time and cumulated change in PHQ-9 values. At each biweekly PHQ-9 measure, we cumulated the number of MOSS app uses. For almost all subjects, we see a constant increase of MOSS app use over time, indicated by the length of arcs between dots of same color.

The scatter plots indicates a negative correlation between cumulated change in PHQ-9 and the total number of MOSS app uses between t_0_ and t_end_.

We conducted a spearman correlation analysis between total app starts and change in PHQ-9 from t_0_ to t_end_ of the 12 subjects classified as clinically depressed at t_0_ and with a system adherence of at least 4 weeks. We observed a negative correlation with rho=-.498 and *P*=.099.

### Depression Detection

**Figure 9:**
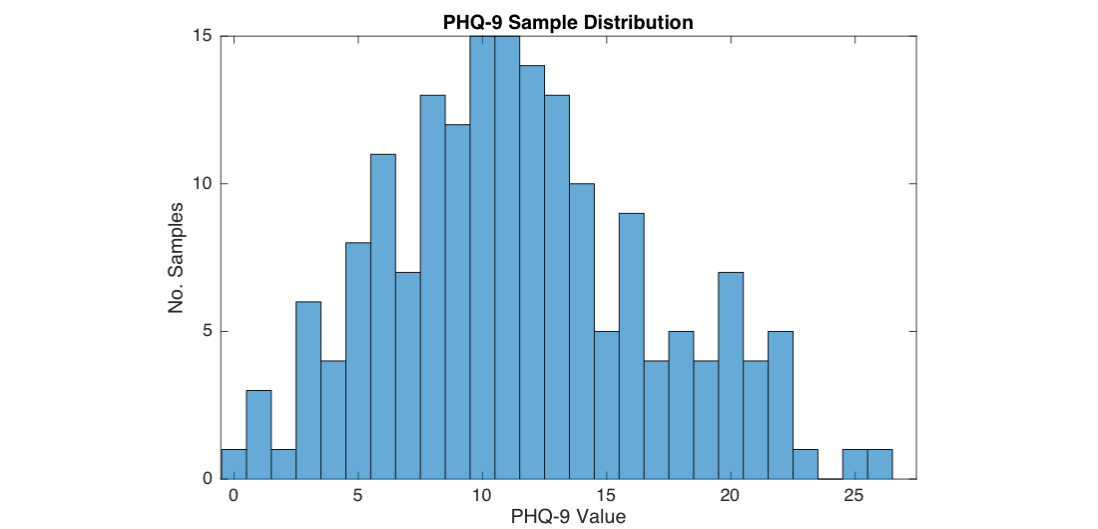
Sample distribution of the 143 Personal Health Questionnaire (PHQ-9) samples of the 36 participating subjects.

Figure 9 shows the sample distribution of the 143 PHQ-9 samples of the 36 subjects with an adherence of at least 2 weeks collected during the trial. Each sample represents a PHQ-9 score provided by a subject via a questionnaire within the MOSS app triggered every 14 days. The distribution shows, that the majority of samples represents a PHQ-9 value close to the classification threshold for clinical depression of 11.

Table 4 shows the average SVM cross-validation score and the RFC out of bag performance with respect to a binary classification of samples with a PHQ-9≥11 and PHQ-9≤10. We separately report sensitivity, specificity, and accuracy. Where sensitivity represents the fraction of samples correctly classified as PHQ-9≥11, specificity represents the fraction of samples correctly classified as PHQ-9≤10, and accuracy represents the fraction of correctly classified samples among both groups. The RFC showed the highest accuracy performance with 61.5 at 450 trees in the model (ntrees = 450). The SVM performed slightly worse with an average accuracy of 59.4. The SVM favored sensitivity over specificity leading to a higher sensitivity score of 72.5 compared with the RFC at 62.3, whereas the RFC has a higher specificity score of 60.8 compared with 47.3.

**Table 4:** Classification performance of support vector machines and random forest classifier.

PHQ-9^a^≥11 vs PHQ-9≤10 classification performance	Support vector machines, radial basis function kernel	Random forest classifier, ntrees = 450
Accuracy	59.4	61.5
Sensitivity	72.5	62.3
Specificity	47.3	60.8

^a^PHQ-9: Personal Health Questionnaire (self-reported depression survey).

## Discussion

### Principal Findings

Based on commonly available smartphone sensor data an array of proxies for physical and social behavior known to be related to a person’s mental health status were introduced. Magnitude of behavior proxies over time periods of 24 hours in comparison to assumptions about healthy behavior were successfully used to dynamically provide meaningful interventions to support people with depressive symptoms in their everyday life. For participants with a clinically relevant PHQ-9 score and an extended MOSS app adherence, a significant drop in PHQ-9 was observed. Among these participants, the relation between frequency of MOSS app usage and change in PHQ-9 scores showed a negative trend. Albeit the fact that we addressed a target population where low motivation toward treatment engagement can be assumed [53], retention rate was above average retention rate of android apps [54].

Two different, supervised, nonlinear machine learning models trained on multiple features calculated from collected sensor data, were able to distinguish between subjects above and below a clinically relevant PHQ-9 score with comparable accuracy exceeding the performance of a random binary classifier.

### Limitations

While this work could present the first app of a context sensitive smartphone app to support people with depressive symptoms, the results are preliminary and a number of limitations need to be addressed. The clinical study carried out is based on a nonrandomized, uncontrolled single-arm study design, which rules out the possibility to prove a direct causal link between symptom improvement and MOSS app use. Additionally, to lower the inhibition threshold, subjects were not asked to provide information about relevant control variables such as other current treatments to rule out their impact on treatment outcome. Furthermore, although research has shown that the PHQ-9 is strongly correlated with depression, not all subjects with an elevated PHQ-9 are certain to have a depression. Moreover, in this first pilot we did not quantify the efficacy of the proposed recommendation algorithm, as this would involve detailed feedback from participants in order to judge appropriateness of context-related intervention recommendations.

### Conclusions

To our best knowledge, this study presents the first trial of a context sensitive smartphone app to support people with depressive symptoms under real life conditions. Although we were able to observe an improvement of subject’s depression levels, evidence in the form of a large RCT needs to be collected. Nevertheless, we assume that the presented approach is a cause for thought for a new generation of digital health interventions, providing caretakers with tools to design context aware and personalized interventions potentially providing a leap forward in the field of digital therapy for people with depression and other mental disorders.

Complementary to the work of Saeb et al [17], we could successfully demonstrate a first proof of concept for the detection of clinically relevant PHQ-9 levels using nonlinear models on features extracted from smartphone sensor data. This includes WiFi, accelerometer, GPS, and phone usage statistics, acting as proxies for physical and social behavior. Albeit the moderate classification performance, the presented work shows yet another promising direction to develop passive depression detection toward clinically relevant levels. Improved models would create opportunities for unobtrusive mental health screening potentially able to alert a subject if a critical mental state is reached and professional treatment highly desirable. In conclusion, this could not only relieve the health care system by preventing severe cases from getting into worse and costlier states but also by preventing subjects with a subclinical PHQ-9 value to strain the system.

## Abbreviations

3D: three-dimensional
CBT: cognitive behavior therapy
CV: cross validation
GPS: global positioning systems
IQR: interquartile range
MOSS: Mobile Sensing and Support
PHQ-9: Personal Health Questionnaire
RBF: radial basis function
RCT: randomized controlled trial
RFC: random forest classifier
SVM: support vector machine
WHO: World Health Organization
